# The Effect of Hydrogen Gas and Water Vapor in Catalytic Chemical Vapor Deposition on the Structure of Vertically Aligned Carbon Nanotubes

**DOI:** 10.3390/ma18235309

**Published:** 2025-11-25

**Authors:** Lilla Nánai, Tamás Gyulavári, Zsejke-Réka Tóth, Zsuzsanna Pápa, Judit Budai, Daniel Koncz-Horvath, Klara Hernadi

**Affiliations:** 1 Institute of Physical Metallurgy, Metal Forming and Nanotechnology, University of Miskolc, 3515 Miskolc, Hungary; 2 Department of Applied and Environmental Chemistry, University of Szeged, Rerrich Béla Sqr. 1, 6720 Szeged, Hungary; gyulavarit@chem.u-szeged.hu; 3 Nanostructured Materials and Bio-Nano-Interfaces Center, Interdisciplinary Research Institute on Bio-Nano-Sciences, Babes-Bolyai University, T. Laurian 42, 400271 Cluj-Napoca, Romania; zsejke.toth@ubbcluj.ro; 4 ELI ALPS, ELI-HU Non-Profit Ltd., 6728 Szeged, Hungaryjbudai@titan.physx.u-szeged.hu (J.B.); 5 Department of Optics and Quantum Electronics, University of Szeged, Dom Sqr. 9, 6720 Szeged, Hungary

**Keywords:** vertically aligned carbon nanotubes, catalytic chemical vapor deposition, pulsed laser deposition

## Abstract

Since the discovery of carbon nanotubes (CNTs), extensive and comprehensive research has been conducted in many areas of materials science. Due to their structural and chemical properties, they can be an important part of electronic devices and structural materials that surround us. In this work, we focused on the preparation and basic analysis of vertically aligned CNTs. An aluminum oxide carrier layer and bimetallic iron–cobalt catalyst layers of different compositions were fabricated on the surface of a silicon substrate using a pulsed laser deposition method. Then, vertically aligned CNTs were grown using a catalytic chemical vapor deposition method based on the thermal decomposition of ethylene. During the experiments, the effect of water vapor and hydrogen gas was investigated on the structure of as-prepared carbon nanotubes. CNT forest samples were characterized by scanning electron microscopy and Raman spectroscopy. One of the most important findings of this research is that the presence of hydrogen gas in the CCVD system is essential, but high-quality vertically aligned CNTs can be produced on silicon substrates even without water vapor.

## 1. Introduction

Significant research results have been obtained in the field of carbon nanotubes (CNTs) since their discovery [1,2]. Today, they have become a fundamental component of materials science and nanotechnology research, mainly because CNTs have outstanding tensile strength, and electrical and conductive properties [3,4,5]. These properties correlate with the purity, structure, and size of CNTs. There are several fabrication methods that allow carbon nanotubes to be synthesized in large quantities with controllable properties to a certain extent, and subsequently commercialized. The most common methods are based on electric arc discharge, laser ablation, and thermal hydrocarbon decomposition [3,6,7,8,9,10].

The synthesis method as well as the materials and parameters used during fabrication drastically influence the structure and properties of CNTs; therefore, their production must be carefully planned according to their subsequent application [11,12,13,14,15,16]. Thanks to their excellent physical and chemical properties, they can be used in many areas: in construction, energy, and automotive industries for developing composite materials, and in environmental protection and green chemistry for creating gas adsorbents, gas sensors, and fuel cells [12,16,17,18,19,20]. CNTs can also constitute an important part of supercapacitors and electrodes. Supercapacitors are promising materials that bridge the gap between electrolytic capacitors and rechargeable batteries [21]. Electrodes must have good conductivity, high temperature stability, long-term chemical stability, high corrosion resistance, and a large surface area per unit volume and weight [21]. Additional requirements include environmental friendliness and low cost. Carbon-based materials (such as graphene and CNTs) may be ideal for meeting the above requirements, which could represent a breakthrough in energy storage [22,23].

Well-oriented CNTs are considered a unique group of CNTs, referred to in the literature as vertically aligned carbon nanotubes (VACNTs) or carbon nanotube forests due to their characteristic structure. Unlike conventional CNTs, these structures can only be produced using a catalytic chemical vapor deposition (CCVD) process based on the thermal decomposition of hydrocarbons (or other carbon-containing precursors). VACNTs were first successfully produced by Wang and his research group using the CCVD method on a silicon substrate surface in 1996 [24]. In 2004, Hata et al. made considerable progress in CNT basic research by producing VACNTs composed of mm-high single-walled CNTs on a silicon substrate [25]. The essential features of this method include the substrate, catalyst, carbon source, a reductive environment (activation of the metal active centers and catalyst nanoparticles), and a high temperature (600–1100 °C, depending on the substrate and carbon source). In addition, a number of other parameters such as catalyst and support layer quality and layer formation conditions, synthesis time, gas flow rates, water vapor quantity, etc., can play an important role in the synthesis [26,27].

The industrial-scale production of VACNTs is currently not feasible, and the optimal conditions for synthesis are unknown [28]. The CCVD method has proved to be a promising synthesis method for producing larger quantities of “defect-free” VACNTs, but the fundamental requirement is to determine the ideal parameters and synthesis conditions. The process is further complicated by the fact that every parameter, beginning with the catalyst layer construction method, through the materials used, to the individual gas flows, has an impact on the structure, composition, and thus the properties of VACNTs.

Previous studies have also examined the effect of hydrogen gas as a substrate pretreatment step prior to the synthesis of CNT forests [29,30,31,32,33,34,35,36,37]. These studies used a single metal catalyst (iron) with various precursors (ferrocene, ferritin, phthalocyanine), layer deposition techniques (electron-beam evaporation, atomic layer deposition, sputtering), gas components (argon, helium as carrier gas; methane, ethyne), substrates (Inconel 600 metallic plate, copper), and synthesis environments (two-zone tube furnace, temperature, gas flow rates, synthesis time). In our previous work, pulsed laser deposition (PLD) was used to form a bimetallic iron–cobalt catalyst layer with a 1:1 ratio on silicon substrates with different layer thicknesses (1–5 nm) [38]. In these experiments, the height and diameter dependence of carbon nanotube forests on the porosity and thickness of catalytic layers was investigated. It is important to note that the focus of these studies differs from the one presented in this manuscript.

Due to the close relationship between these parameters, much of the basic research to date has focused on optimizing the production of VACNTs. They have similar properties to those of simple CNTs, but thanks to their ordered structure, they have even better electrical and thermal conductivities. For this reason, their future applications are expected to focus on electronic and microelectronic devices (such as high-capacity capacitors, solar cells, photodiodes, electrodes, and gas sensors) and on composite materials for enhanced performance [39,40,41,42,43].

In this work, VACNTs were grown on silicon, one of the most frequently used substrates, and the effect of water vapor and hydrogen gas on the growth and structure of the resulting CNTs was investigated. The effect of catalyst composition was also evaluated by using a bimetallic iron–cobalt catalyst in five different compositions. The catalyst layer was deposited via PLD. The as-prepared samples were characterized by scanning electron microscopy (SEM), transmission electron microscopy (TEM) and Raman spectroscopy.

## 2. Materials and Methods

### 2.1. Materials

The characteristics of the materials used are summarized in Table 1.

### 2.2. Layer Construction and CCVD Synthesis

**Figure 1:**
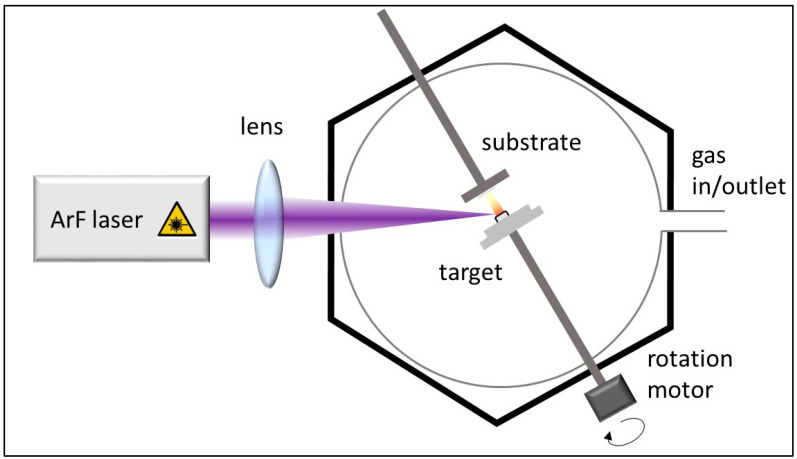
Schematic diagram of pulsed laser deposition (PLD) layer construction method.

Prior to the CCVD experiments, aluminum oxide and iron–cobalt catalyst layers of various compositions were deposited onto the surface of the silicon substrate using the PLD method (Figure 1). To prepare the Al_2_O_3_ support layer and catalyst layers of various iron–cobalt compositions the laser pulses from an ArF excimer laser (LLG TWINAMP, Göttingen, Germany, wavelength 193 nm, pulse length 18 ns, repetition rate 10 Hz) were focused onto pressed targets. The average fluence was 13 J/cm^2^. The pulse energy variation during an experiment was ~10%. The support and catalyst layers were fabricated with 3000 and 600 laser shots, respectively. In case of the support layer 1 Pa oxygen background, while in case of the 5 nm thick catalyst layers 0.5 Pa argon background was set in each case. Detailed description of the layer deposition can be found elsewhere [38,44].

**Figure 2:**
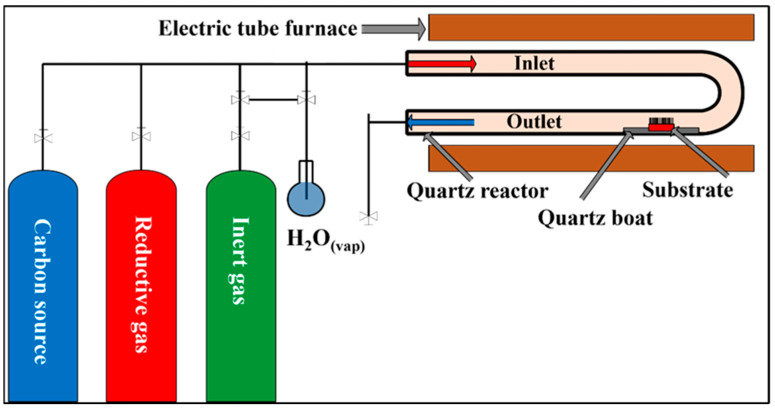
Schematic representation of the CCVD system.

The thickness of the catalyst layers was determined by ellipsometry measurements, yielding a value of approximately 5 nm. During the experiments, five different iron–cobalt (Fe:Co = 1:3, 2:3, 1:1, 3:2, and 3:1) catalyst ratios were applied. Prior to synthesis, once the substrate and catalyst layers had been formed, the silicon sheets were cut into 0.5 × 0.5 cm pieces so that they could be properly positioned in the quartz boat used during synthesis. The experimental setup for the synthesis was based on the thermal decomposition of ethylene using a CCVD method, as shown in Figure 2.

For the synthesis of VACNTs on a silicon substrate, we used a synthesis temperature of 750 °C and controlled the flow rate of the gases in the system with rotameters. Nitrogen (40 cm^3^/min) was used as the carrier gas, while hydrogen gas (80 cm^3^/min) was used to provide a reductive environment. Ethylene (110 cm^3^/min) served as the carbon source, and water vapor (38 cm^3^/min) was used to activate the catalyst particles and remove the amorphous carbon produced during synthesis. The water vapor was introduced into the reaction chamber via a bubbling method through the nitrogen branch. The flow rates were varied only in the experimental series where the effect of these parameters on the structure of CNTs was investigated. In the initial stage of the synthesis, the system was purged with nitrogen gas through the system for 5 min, then hydrogen gas was applied to reduce the catalyst layer. After an additional 5 min, the flow of ethylene and water vapor simultaneously started. The reaction time for CCVD syntheses was 25 min.

### 2.3. Characterization of VACNTs

The samples were characterized using a SEM and Raman spectroscopy. The electron microscope provided information about the structure and height of the carbon nanotubes consisting of the CNT forests. The measurements were performed using a Hitachi S-4700 Type II FE-SEM scanning electron microscope operated at an acceleration voltage of 10 keV. During the SEM imaging, the sample holder was tilted at an angle of 35° to observe the sides of the VACNTs and determine their height. ImageJ software (version 1.43, released April 2010) was used to analyze the images. To determine the quality of the CNTs and their graphitic properties, a Thermo Scientific DXR Raman spectrometer was applied, which was operated with an excitation laser (λ = 532 nm) at a power of 5 mW.

## 3. Results and Discussion

VACNTs were synthesized using the CCVD method on the surface of a silicon substrate covered with an aluminum oxide carrier layer. On the carrier layer, catalyst layers of different compositions (1:3, 2:3, 1:1, 3:2, 3:1) were deposited using the PLD method. In a series of experiments, we investigated the effect of water vapor and hydrogen gas on the CNT forest structure, including their height and the quality of CNTs at different catalyst ratios. We also examined the combined effect of these varying parameters.

### 3.1. Investigation of CNT Forests Synthesized in the Presence of Water Vapor and Hydrogen

Both water vapor and hydrogen gas play an important role in the production of VACNTs. Hydrogen gas reduces the bimetallic catalyst layer of iron and cobalt oxide deposited by the PLD method. During the reduction phase, individual iron–cobalt catalyst nanoparticles are formed on the substrate surface from the uniform catalyst layer, and these particles become the active centers for VACNT growth [45]. The gaseous carbon source is adsorbed on the surface of the individual catalyst particles, where the C–H and C–C bonds are broken by the high temperature and the catalyst, and the resulting carbon is dissolved into the catalyst particle. Subsequently, upon reaching a certain saturation state, the carbon precipitates on the “colder” metal surface and, through self-assembly, forms an energetically stable, cylindrical CNT [45,46,47]. The thermal decomposition of hydrocarbons and the temperature gradient between carbon precipitation and self-assembly drive the further growth of CNTs until the catalyst particles are deactivated during the process. This long-standing concept was confirmed in a recent publication, where we used a theoretical approach to verify the molecular processes that are presumed to occur on the surface of alumina supports under the conditions used in CCVD [45].

Water vapor is present in the system as a mild oxidizing agent, whose task is to remove the amorphous carbon produced during synthesis from the synthesis chamber without damaging the CNTs, thereby restoring the catalyst particles and increasing their lifetime and prolonging the activity [25]. The quality and height of the resulting CNTs can be significantly improved by adding the proper amount of water vapor, but it is important to determine the optimum amount.

**Figure 3:**
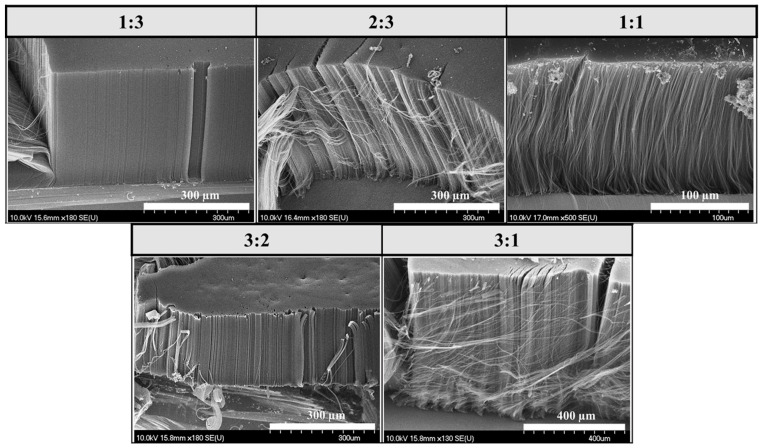
SEM images of samples synthesized on silicon substrates coated with an aluminum oxide support layer, with different iron–cobalt catalyst compositions, in the presence of water vapor and hydrogen gas.

In this series of experiments, both water vapor and hydrogen gas were present in the CCVD system, and the results presented later in this article were always compared to this reference sample series (Figure 3).

Based on the SEM images, uniformly distributed, well-structured, dense, and VACNTs were formed on the surface of the silicon substrate in all cases. Based on the results, it can also be concluded that the combination of water vapor and hydrogen gas did indeed result in the formation of well-structured, VACNTs during synthesis. The height of the CNTs will be shown in Figure 7, comparing the heights of the samples prepared in the three-sample series. The average height of the VACNTs was 0.57 mm. However, contrary to the results reported in the literature [33,48,49], these experiments did not produce the tallest and best-aligned CNTs at a 1:1 iron–cobalt catalyst ratio; in fact, in our studies, these were the lowest VACNTs in the series, with a height of 0.24 mm.

### 3.2. Investigation of CNT Forests Synthesized in the Presence of Hydrogen Without Water Vapor

**Figure 4:**
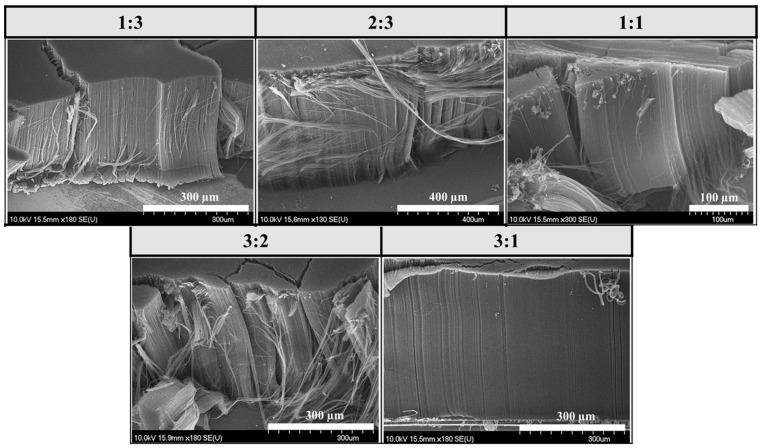
SEM images of samples synthesized on silicon substrates coated with an aluminum oxide layer, with different iron–cobalt catalyst compositions, in the presence of hydrogen gas and without water vapor.

In this series of experiments, it was investigated how the height and structure of VACNTs change in the CCVD system without water vapor, i.e., using only carrier gas, carbon source, and hydrogen gas (Figure 4).

**Figure 5:**
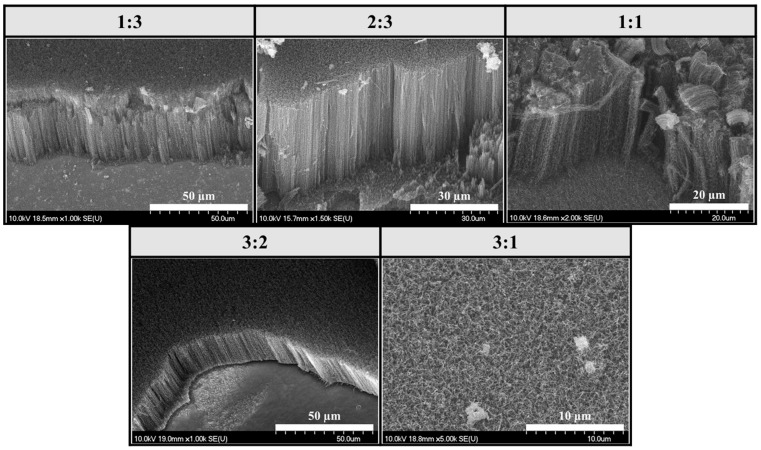
SEM images of samples synthesized on silicon substrates coated with an aluminum oxide carrier layer, with different iron–cobalt catalyst compositions, in the presence of water vapor, without hydrogen gas.

SEM images show that, in all cases, the VACNTs formed during the synthesis exhibit uniform height and a dense distribution. As in the first series, the lowest CNT forests, with a height of 0.36 mm, were formed at a catalyst ratio of 1:1 iron–cobalt again. Although slightly taller, VACNTs with an average height of 0.62 mm were formed in this series. High-resolution SEM images revealed that the orientation of the CNTs was not as good as in the VACNTs produced in the first sample series (Figure 5). Based on these findings, it can be concluded that slightly taller VACNTs were formed in the absence of water vapor, since, in general, the water vapor in the system begins to degrade not only the amorphous carbon but the (less graphitic) outer walls and ends of the CNTs too to some extent, thus decreasing the height. Furthermore, in the absence of water vapor, the walls of CNTs have more defect sites because the healing effect of water vapor cannot occur.

### 3.3. Investigation of CNT Forests Synthesized in the Presence of Water Vapor Without Hydrogen

**Figure 6:**
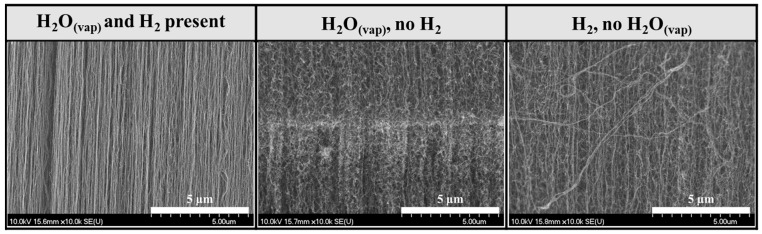
High-resolution SEM images of samples synthesized with iron–cobalt ratio 2:3 catalyst composition in three sample series.

In the last sample series, it was investigated how the height and structure of VACNTs change in the CCVD system without hydrogen gas, i.e., in the presence of only carrier gas, carbon source, and water vapor (Figure 6).

**Figure 7:**
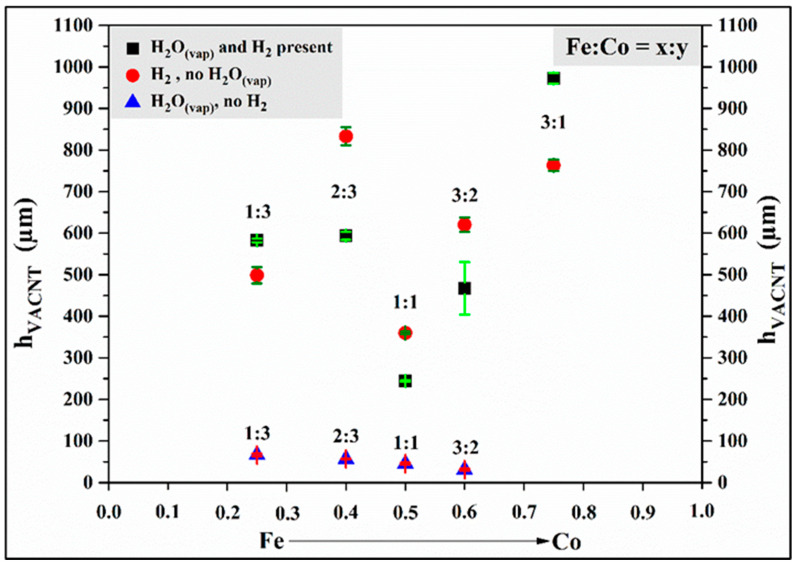
Distribution of VACNT heights produced in three sample series.

Based on the SEM images, a remarkable difference can be observed compared to the previous samples from the other two series. Although the characteristic structure of VACNTs could be observed at all ratios except at iron–cobalt 3:1 composition, the height and orientation of the CNTs were less prominent and defined than in the other samples, respectively. (Repeating the synthesis of the 3:1 sample did not yield different results.) This sample series clearly highlights the role of hydrogen gas in CCVD synthesis. Since there was no hydrogen gas in the system during the initial stage of synthesis, the transformation and activation of the catalyst layer did not occur due to the lack of a reductive environment. In the subsequent stage of synthesis, ethylene and water vapor flowed into the system simultaneously, so it can be presumed that they together replaced the role of hydrogen gas. During the thermal decomposition of ethylene, the hydrogen produced by the breaking of chemical bonds may have contributed to the creation of a reducing environment, and water vapor continued to activate the catalyst particles. However, these processes took place slowly, so the growth of CNTs was inhibited for most of the 25-min-long synthesis. Furthermore, it can be assumed that the water vapor in the system oxidized the existing CNTs, further reducing their height (Figure 7).

### 3.4. Comparison of Samples

In this subchapter, the results of the three experimental series will be compared and supplemented with observations and conclusions drawn so far.

First, the differences in the quality and orientation of CNTs will be highlighted based on comparative high-resolution SEM images (Figure 6). Since similar observations were made for all iron–cobalt catalyst ratios, only SEM images of the samples with a 2:3 iron–cobalt composition are presented.

High-resolution SEM images show that the orientation and quality of CNTs significantly worsened in samples synthesized without water vapor and hydrogen gas. A further sharp decrease in quality was observed in samples synthesized without hydrogen gas.

Based on the results presented in the previous subchapters, it was already concluded that, contrary to the results reported in the literature [33,48,49], the lowest VACNTs were obtained in the experiments with a 1:1 iron–cobalt ratio. When comparing the heights of the samples produced in this series, no clear trend can be observed related to changes in catalyst composition (Figure 7).

Based on the changes observed in the VACNTs produced in the three experimental series as a function of catalyst composition, it can be stated that the highest nanotubes were produced at iron–cobalt catalyst ratios of 3:1 and 2:3, while the lowest VACNTs were produced at iron–cobalt ratios of 1:1 and 3:2. It can also be assumed that the height of the CNT forests synthesized in the absence of water vapor is higher than that of the samples in the first experimental series (in the presence of water vapor and hydrogen gas) because, without water vapor, the mild oxidative environment that could reduce the height of the carbon nanotube forests did not occur in the system. As a result, however, the quality of the CNTs was slightly poorer (see Raman results), containing more irregularities, which indicated the appearance of defect sites. Thus, it can be concluded that relatively high-quality VACNTs can be produced without water vapor, but not without hydrogen gas, so the reductive environment created in the CCVD system by the hydrogen gas is essential for the final performance of the synthesis.

**Figure 8:**
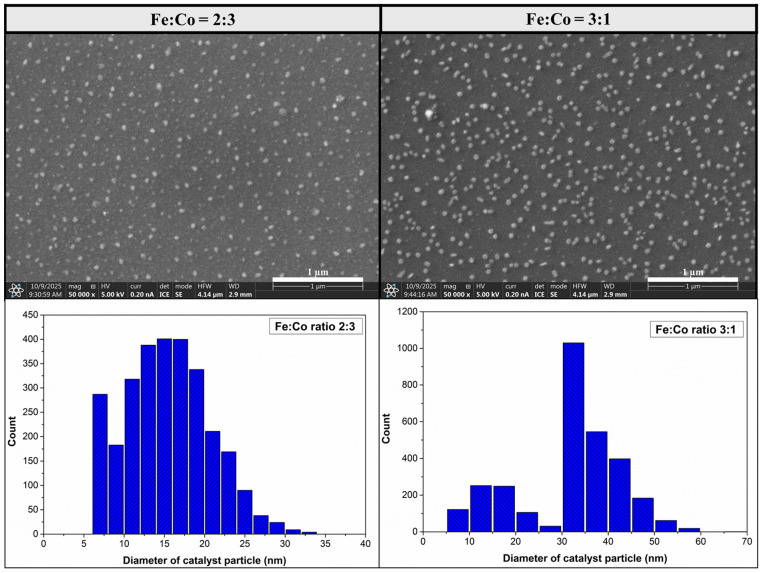
SEM images of blank synthesis on Si substrate, with the composition iron cobalt catalyst 2:3; 3:1 and their histograms of catalyst particle size distribution.

To obtain further information about the catalyst layer, blank synthesis (Figure 8) was performed on iron–cobalt catalyst layers with ratios of 3:1 and 2:3, which showed to be superior to the others based on the results presented so far (Figure 3, Figure 4 and Figure 7). The blank synthesis differed from the experimental description presented previously in that only nitrogen and hydrogen gas were present in the system, the synthesis time was 5 min, and the temperature remained at 750 °C. Based on blank synthesis, average catalyst particle size with iron–cobalt 2:3 ratio was from 7 nm to 23 nm ± 5.4 nm, and with iron–cobalt 1:3 ratio it was from 31 nm to 41 nm ± 8.1 nm.

**Figure 9:**
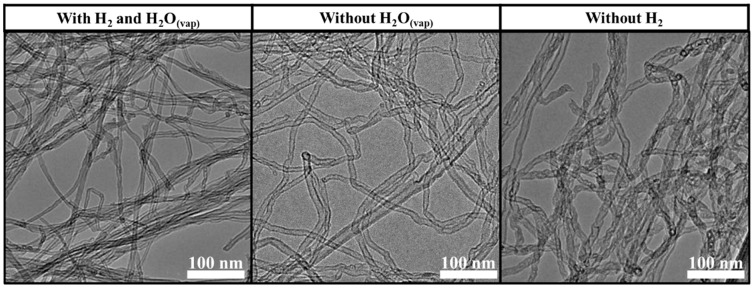
TEM images of carbon nanotubes from each of the three experimental series presented earlier (with hydrogen gas and water vapor, without water vapor and without hydrogen gas).

TEM images were taken of one sample from each of the three experimental series presented earlier (Figure 9). These results confirmed our previous observations. Based on the TEM images, the sample produced without hydrogen gas had the most structural irregularities or defects, while the sample produced without water vapor formed quite straight carbon nanotubes with a few structural defects here and there. CNTs synthesized in the presence of water vapor and hydrogen gas exhibited the fewest structural defects. Based on the analysis of TEM images, the outer diameter of the carbon nanotubes was evaluated to be between 12 and 16 nm on average.

### 3.5. Raman Spectroscopy Observations

**Figure 10:**
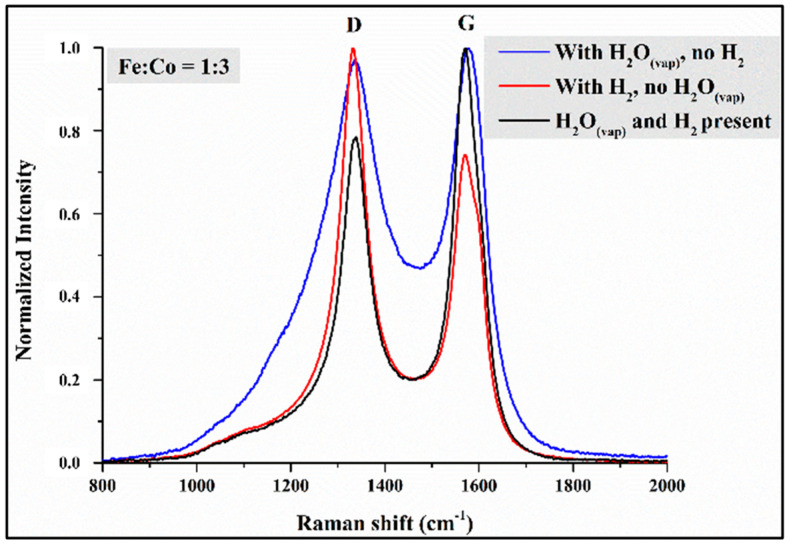
Raman spectra of samples synthesized with an iron–cobalt catalyst ratio of 1:3 in three sample series.

During Raman spectroscopy measurements, we wanted to obtain information about the graphitic properties of the VACNTs produced based on the intensity ratios of the D and G peaks characteristic of carbon-containing materials (Figure 10). Since the results did not reveal any significant trends in the graphitization of CNT forests or changes in catalyst composition, only the Raman spectra of samples with a 1:3 iron–cobalt ratio are presented (Figure 10). The spectra are normalized for easier comparison and interpretation.

Based on the shape and intensity of the D and G peaks (Figure 10), differences can be observed between the samples produced with a 1:3 iron–cobalt ratio in the three series of experiments. Based on the determined I_D_/I_G_ intensity ratio values, it was concluded that the sample synthesized without water vapor had the most structural defects (I_D_/I_G_ = 1.35), meaning that the resulting high CNTs did not have outstanding graphitic properties. The CNT sample synthesized without hydrogen gas using a 1:3 iron–cobalt catalyst did not have an outstanding structure based on the SEM image in Figure 5. However, based on the Raman spectrum (Figure 10), fewer structural defects formed in their structure, resulting in better graphitic properties (I_D_/I_G_ = 0.97) than in the sample without water vapor. Based on the measurements, the reference sample series produced the best-structured CNT forests, which had excellent graphitization (I_D_/I_G_ = 0.79).

Due to the outstanding height of the VACNTs grown on the silicon substrate, the Raman spectra were recorded at three different points: at the bottom of the CNT forests, close to the substrate; in the middle of the CNT forests; and at the top of the CNT forests. Based on the measurements taken in this way, we wanted to obtain information on how the quality of CNTs changes as the synthesis progresses. Similarly to our previous work [35], we found that the I_D_/I_G_ ratios showed a decreasing trend from the bottom to the top of the CNTs. It can be concluded that at the beginning of the CCVD synthesis, the structure of CNTs contains fewer defects, i.e., it is more graphitic, and as the synthesis progresses and the activity of the catalyst particles decreases, the number of defects in the structure of CNTs increases.

Based on the results of Raman spectroscopy measurements (Figure 10), it was hypothesized that the CNTs forming and growing on the catalyst particle during CCVD synthesis follow the root mechanism. That is, a strong interaction develops between the silicon substrate and the iron–cobalt catalyst, causing the catalyst particle to remain on the substrate surface throughout the synthesis. The change in quality along the length of the CNTs can be explained by the fact that at the beginning of the synthesis, meaning the top of the CNTs, high-quality, defect-free structures are formed because the catalyst particles are still “fresh” and have high activity and selectivity. As the synthesis progresses, i.e., as we move toward the bottom of the CNTs, the structure of the VACNTs contains more and more defects, which can be explained by the decrease in the selectivity and activity of the catalyst nanoparticles.

## 4. Summary

The PLD technique was used to form well-distributed iron–cobalt catalyst layers on the surface of a silicon substrate with uniform thickness. During the CCVD synthesis conducted at 750 °C, high-quality CNTs with a high degree of vertical alignment were successfully produced. During the experiments, the effect of the catalyst composition, water vapor, and hydrogen gas was studied on the structure and properties of the CNTs. Based on SEM images and Raman spectroscopy measurements, it was concluded that the presence of hydrogen gas in the CCVD system is essential, but without water vapor, high-quality VACNTs can be still produced on a silicon substrate. The structure of CNTs contains an increasing number of defects as synthesis progresses, i.e., their graphitic properties worsen from the top to the bottom of the VACNTs. Contrary to the results reported in the literature, the lowest VACNTs were formed in the presence of a 1:1 iron–cobalt catalyst composition. Finally, the results presented here also clearly illustrate the close relationship between the parameters used in the production of VACNTs by CCVD based on the thermal decomposition of hydrocarbons, which is why so much basic research continues to focus on optimizing synthesis parameters to this day.

## Figures and Tables

**Table 1:** Materials used during the experiments.

Role of Material	Type of Material	Purity	Distribution Company
Substrate	silicon wafer	p-type (100) 0.8 mm	WRS Materials (San Jose, CA, USA)
Support layer	aluminum oxide	99.99%	MERCK (Darmstadt, Germany)
Catalyst layer	cobalt(II) oxide	99%	ALDRICH (Taufkirchen, Germany)
	iron(III) oxide	99.9%	ALDRICH (Taufkirchen, Germany)
CCVD gas feed	ethylene	≥99.9%	MESSER (Budapest, Hungary)
	hydrogen	99.5%	MESSER (Budapest, Hungary)
	nitrogen	99.9%	MESSER (Budapest, Hungary)

## Data Availability

The original contributions presented in this study are included in the article. Further inquiries can be directed to the corresponding authors.
